# P04 Primary mediastinal large B-cell lymphoma in a Caucasian female with anti-SAE-1 positive dermatomyositis

**DOI:** 10.1093/rap/rkab068.003

**Published:** 2021-10-19

**Authors:** Yvonne Chang, Eleni Stathopoulou, Rehab Mohammed, Jon Goulding

**Affiliations:** 1 Rheumatology, University Hospitals Birmingham NHS Foundation Trust, Solihull, United Kingdom; 2 Dermatology, University Hospitals Birmingham NHS Foundation Trust, Solihull, United Kingdom

## Abstract

**Case report - Introduction:**

This is a case of a female patient who was diagnosed with dermatomyositis with anti-Small ubiquitin-like modifier Activating Enzyme-1 (SAE-1) and anti-Ro52 autoantibodies. She had extensive skin involvement with mild extracutaneous features and responded well to a combination of prednisolone, hydroxychloroquine and azathioprine. She subsequently developed primary mediastinal large B-cell lymphoma about 3 years after diagnosis of dermatomyositis.

**Case report - Case description:**

A 59-year-old Caucasian female presented in January 2018 to the dermatologist with a 6-month history of itchy, dry, flaky skin around her eyes with periorbital erythema which later progressed to fixed periorbital oedema. At that point, a diagnostic biopsy was taken from the left lower eyelid which showed a deep inflammatory infiltrate with significant oedema and dilated blood vessels. A few weeks later, she developed a widespread erythematous rash. Other than some cramps and myalgia in the thighs, there was no muscle weakness or dysphagia. There were no red flag symptoms, but some dry cough without dyspnoea. Clinical examination revealed periungual erythema, periorbital erythema and oedema, widespread erythematous eruption on the torso and limbs and papular eruptions on the hand dorsum. Muscle power in upper and lower limbs scored 5 in the MRC scale. Blood tests showed slight lymphopaenia, antinuclear antibodies titre 1:400, dsDNA and ENA negative, raised IgG kappa monoclonal protein at 10g/L with normal kappa/lambda ratio, creatine kinase initially 93 that later rose to 209, slightly raised lactate dehydrogenase 262, otherwise unremarkable biochemistry and inflammatory markers. Extended myositis screen with immunoblot revealed positive SAE-1 and anti-Ro52 autoantibodies. MRI of the femurs showed mild oedema in the adductors. Electromyogram showed some myopathic motor units, not florid but convincingly present. Computed tomography of neck, thorax, abdomen and pelvis did not reveal any underlying malignancy at the time. The diagnosis of SAE-1-positive dermatomyositis was made and she was treated with high-dose prednisolone (1mg/kg), azathioprine and hydroxychloroquine with good response and dermatomyositis went into remission. In December 2020, she developed dyspnoea and was found to have left-sided pleural effusion and anterior mediastinal mass, a biopsy of which confirmed primary mediastinal large B-cell lymphoma. She is currently receiving chemotherapy with rituximab-cyclophosphamide-doxorubicin-vincristine-prednisolone and azathioprine was discontinued. Her dermatomyositis remains in remission.

**Case report - Discussion:**

The SAE-1 autoantibody was first identified in 2007. Its prevalence ranges from 1% to 8% in different cohorts. The main clinical feature of anti-SAE-1-positive disease tends to be a widespread cutaneous involvement which is typically pruritic, either amyopathic or associated with mild muscle involvement. Other extracutaneous manifestations include arthralgia, dysphagia and interstitial lung disease (ILD) with variable frequency in different cohorts. ILD is usually milder than in other amyopathic dermatomyositis subgroups.

Our patient presented with rather extensive cutaneous disease (clinical photography available) requiring high-dose prednisolone and we decided to add azathioprine as a steroid-sparing agent. She had minimal muscle disease and no other extracutaneous manifestations. Interestingly, she developed lymphoma 3 years following the diagnosis of dermatomyositis.

The association between dermatomyositis and malignancy is well-established. The generally accepted definition of cancer-associated myositis is malignancy within 3 years of disease onset. Dermatomyositis as opposed to polymyositis, increasing age, male sex, dysphagia, cutaneous ulceration and the presence of anti-transcription intermediary factor-1 gamma antibodies (TIF1γ) were all associated with increased cancer risk in a recent meta-analysis by Oldroyd et al. Anti-SAE-1 is variably reported to be associated with cancers. A recently published study of a North American cohort of anti-SAE-1-positive dermatomyositis patients reported five cases of cancers, mainly internal malignancies and one case of B-cell lymphoproliferative disorder. We are not aware of any more reported cases of lymphoma in patients with anti-SAE-1 autoantibodies. A 2018 study did report two cases of dermatomyositis patients who developed lymphoma without specifying the autoantibody profile in those patients.

Our case contributes to the fact that dermatomyositis can be associated with malignancies. It also raises awareness that this rare subgroup of dermatomyositis with SAE-1 autoantibodies can be associated with lymphoma. However, this association has not been widely reported in the literature.

**Case report - Key learning points:**

- We need to consider whether the immunosuppressive medications used to treat dermatomyositis could be a contributing factor to the development of cancers in these patients. As the mechanism of the association between malignancies and inflammatory myositis is unclear and may be multifactorial, it is worth noting that our patient was treated with azathioprine. There are several studies showing increased risk of lymphoma in patients with inflammatory bowel disease treated with azathioprine or thiopurines in general.
- Immunosuppression could also lead to Epstein-Barr virus-associated lymphoma. In our case, Epstein-Barr virus antibodies have not been checked.
- We should remain vigilant to the possible development of cancer in dermatomyositis patients within 3—5 years before or after disease onset. In addition to the routine national screening guidelines, computed tomography of thorax, abdomen and pelvis may be useful in early detection of cancer. However, there is no clear consensus on whether there is a need for follow-up malignancy screening and how often we should screen in the subsequent years post-diagnosis.
- It is recommended that at follow-up appointments, we should take a thorough history including red flag symptoms and conduct a detailed clinical examination of the patients.
- There is definitely a need for more studies and consensus on setting guidelines to establish screening strategies for malignancies in patients with dermatomyositis.

